# The Effect of Immunosuppressive Adjuvant Kynurenine on Type 1 Diabetes Vaccine

**DOI:** 10.3389/fimmu.2021.681328

**Published:** 2021-07-07

**Authors:** Jing Sun, Jiandong Shi, Jianfang Li, Meini Wu, Yanhan Li, Sengquan Jia, Chunli Ma, Xinyi Wang, Zhiyuan Li, Ningzhu Hu, Yunzhang Hu

**Affiliations:** ^1^ Institute of Medical Biology, Chinese Academy of Medical Sciences and Peking Union Medical College, Kunming, China; ^2^ Kunming Medical University, Kunming, China

**Keywords:** immunosuppressive, adjuvant, kynurenine, vaccine, Type 1 diabetes vaccine

## Abstract

Inducing antigen-specific tolerance is a promising treatment for preventing or reversing Type 1 diabetes (T1D). In contrast to a vaccine that induces immune responses against pathogens, a tolerogenic vaccine can suppress immunity against antigens causing diseases by administrating a mixture of self-antigens with an adjuvant that decreases the strength of antigen-specific response. Kynurenine (Kyn) is an endogenous substance that can inhibit the natural killer cell and T cell proliferation and promote the differentiation of naïve T cells into regulatory T cells (T_regs_). In this study, we evaluated the efficacy of Kyn as a novel suppressive adjuvant. Kyn was co-immunized with GAD65 phage vaccine to induce T_reg_ cells and tolerogenic responses for the prevention of T1D in NOD mouse model. Mice were subcutaneously immunized two times with 10^11^ Pfu (100μL,10^12^ Pfu/ml) GAD65 phage vaccine doses mixed with 200 μg of Kyn. Serum antibodies and cytokines were detected by ELISA and electrochemiluminescence, respectively. Flow cytometry assay was used to analyze DC and Treg. MTS was used for the analysis of spleen lymphocyte proliferation. RNA sequencing was used to investigate mRNA and miRNA expression profiles in spleen lymphocytes. Compared to GAD65 phage vaccine alone, co-immunization of Kyn and GAD65 phage vaccine resulted in the prevention of hyperglycemia in 60% of mice for at least one month. Further, Kyn enhances GAD65-specific Th2-mediated immune responses; regulates the Th1/Th2 imbalance and increases the secretion of Th2 cytokines and the number of CD4^+^CD25^+^Foxp3^+^T cells; suppresses DC maturation and GAD65-specific T lymphocyte proliferation. Moreover, we integrated Kyn related miRNA and mRNA expression profiles obtained from the spleen lymphocyte RNA-sequencing which was stimulated by Kyn *in vitro*. These data provide an important basis for understanding the mechanisms underlying Kyn as an immunosuppressive adjuvant which regulated the immune response. These findings suggest that Kyn can serve as an effective suppressive adjuvant candidate for Type 1 diabetes vaccines.

## Highlights

• Co-immunization of Kyn and GAD65 phage vaccine resulted in prevention of hyperglycemia in 60% of mice for at least one month.
• Co-immunization of Kyn and GAD65 phage vaccine enhances GAD65-specific Th2-mediated immune responses; regulates the Th1/Th2 imbalance and increases the secretion of Th2 cytokines and the number of CD4^+^CD25^+^Foxp3^+^ T cells; suppresses DC maturation and GAD65-specific T lymphocyte proliferation.
• RNA sequencing provides an important basis for understanding the mechanisms underlying Kyn as an immunosuppressive adjuvant which regulated the immune response.

## Introduction

Type 1 diabetes (T1D) is an autoimmune disease attributed to the immune-mediated progressive destruction of *β* cells in the pancreatic islets, which results in hyperglycemia. Autoantibodies against insulin, including 65 kDa glutamic acid decarboxylase (GAD65), insulinoma-associated protein 2 (IA-2), and zinc transporter 8 (ZnT8), are proteins associated with secretory *β*-cell granules. It can be used as biomarkers of T1D-associated autoimmunity. These proteins can be identified months to years before the onset of T1D and served as developing risk markers (1, 2).

Infiltration of islet antigen-specific T cells, activation of pro-inflammatory antigen-presenting cells, and loss of Foxp3^+^ regulatory T cells (Tregs) are three of the most T1D characteristic immunopathological processes (3).

Nearly 20 million people suffer from T1D worldwide. Although life-long insulin treatment can alleviate symptoms and delay organ damage, it does not reverse the antigen-specific T cell responses toward *β* cells. Therefore, a novel treatment strategy is required to improve therapeutic effects. Some scholars (4) believe that *β* cell autoantigens presented in non-inflammatory contexts can regulate auto-reactive T cells and generate *β* cell protection. Recovering antigen-specific tolerance or down-regulating the immune response to non-harmful antigens is a promising way to treat T1D.

GAD65 is a major autoantigen in T1D. T-cell reactivity and autoantibodies against GAD65 are early markers of this autoimmune disease process. GAD antibodies have been found in nearly 70–80% of T1D patients at the time of diagnosis (5). Preclinical studies have demonstrated that the administration of the isoform GAD65 in non-obese diabetic (NOD) mouse model can prevent autoimmune destruction of pancreatic *β*-cells. In nearly 80% of NOD mice, long-term normoglycemia was restored by repeated administration of GAD65-alum. Moreover, the injection of aluminum salts of GAD65 (GAD65-Alum) in mice has been shown to reduce GAD-specific Th1 Teff cells (6, 7). Also, GAD65-Alum (Diamyd^®^) has been recently tested in phase II and III clinical trials (8–11), showing drug safety. In addition, Diamyd^®^ appeared to be superior to placebo in preserving residual *β*-cell function at 12–15 months. Moreover, an expansion of IL-10^+^CD4^+^ T cells was observed, indicating the regulatory compartment’s induction (12). Despite these vaccines’ success in mouse models, these vaccines could not induce CD4^+^Foxp3^+^ Tregs that can balance between beneficial and harmful effects of inflammation.

Regulatory T (Treg) cell is believed to have a key role in preventing autoimmunity. Animal studies have illustrated that CD4^+^Foxp3^+^ Tregs can induce tolerance by suppressing the functions of Th1 cells and DCs or by releasing inhibitory cytokines such as TGFβ or IL-10 (13), and form the primary mechanism of peripheral tolerance (14). The most promising immunotherapy for autoimmune disease treatment in humans is autologous Treg cell therapy (15). Some studies (16) have shown that dexamethasone and rapamycin (rapa) could significantly increase cell numbers and function CD4^+^CD25^+^ Treg cells in animal models. However, these immunosuppressive drugs have side effects, such as induction of infection and tumor formation.

Nowadays, many kinds of autoantigen-specific T1D trials involving oral and nasal insulin or recombinant human GAD65 formulated with alum have been continuously applied (17). However, no suitable immunosuppressive drug or immunosuppressive adjuvant can be used in combination with it. Therefore, finding new immunosuppressive drugs or immunosuppressive adjuvants is a key factor for the success of this treatment strategy.

Kynurenine (Kyn) is a tryptophan metabolite produced through tryptophan-2, 3-dioxygenase (TDO) degradation in the liver under physiological conditions (18), and through the indoleamine 2, 3 dioxygenase (IDO) in the extrahepatic tissues including blood and lymph tissue during infection, inflammation, or oxidative stress (19, 20). It has been demonstrated that Kyn activates the cytosolic aryl hydrocarbon receptor (AHR) in a ligand-receptor manner (21), and endogenously regulates systemic inflammation and tolerance. Additionally, AHR has been implicated in various immune functions, including reduce the activity of natural killer (NK) cells (22), inhibit the NK cell and T cell proliferation (23), and promote the differentiation of naïve T cells into Treg instead of into Th17 cells through preventing dendritic cell maturation (24), which supports the role of AHR as an important player in determining the T cell differentiation (25). Because the Kyn-AHR axis has an effect on the proliferation of Tregs, it has been considered a potential therapeutic target for the treatment of autoimmune disorder. In our previous studies, we found that Kyn can serve as an effective suppressive adjuvant for vaccines. Otherwise, Kyn is an endogenous substance that is safer than exogenous substances when considered as a vaccine adjuvant.

In this study, Kyn was co-immunized with GAD65 phage vaccine to induce Treg cells and tolerogenic responses for prevention of T1D in the NOD mouse model. We provided direct evidence that Kyn, as a novel suppressive adjuvant, promotes Foxp3^+^ Treg induction, suppresses dendritic cell maturation and GAD65-specific T cell proliferation, and significantly increases IL-10, IL-4 and TGF-*β*1, decreases of IFN-*γ* and IL-2 in the NOD mouse model. We also analyzed the molecular information provided by transcriptome sequencing of mRNA and miRNA in an *in vitro* Kyn assay, providing a new understanding of the underlying immune response mechanism and a new idea for the development of suppressive adjuvants.

## Materials And Methods

### GAD65 Phage Vaccine Preparation

The recombinant GAD65 phage vaccine expressing the 190–320 amino acid sequence of huGAD65 (GenBank: M81882.1) was constructed in the T7 phage display system by our laboratory. The huGAD65 gene shares 95% amino-acid identity and 98% conservation with mGAD65 (26), respectively. Briefly, we inoculated 50 μl 10^11^ pfu/ml GAD65 phage into 5 ml fresh *Escherichia coli strain* BLT5403 with OD600 = 0.6–0.8, cultured at 37°C and 150 rpm for 3 h. The cultures were diluted 50-fold into 1,000 ml of *Escherichia coli strain* BLT5403 with OD600 = 0.6–0.8, cultured at 37°C and 150 rpm for 3–6 h. Bacteria were collected by centrifugation (30 min at 5,000 rpm.) The supernatant was mixed in 1:5 volume solution containing 20% polyethylene glycol 8000 (PEG-8000) and 2.5 M NaCl, then kept at 4°C overnight to precipitate the phage particles. After that, the precipitate was collected by centrifugation (30 min at 10,000 rpm.), dissolved in 4 ml of phosphate-buffered saline (PBS), and centrifuged at 10,000 rpm for 30 min. The phage particles were then purified by sequential centrifugation of the PBS phage suspension at 10,000 rpm for 30 min. The precipitate was then dissolved in 1 ml of 50 mM Tris-HCl buffer, pH 7.5. Then, an ultrafiltration membrane with a molecular weight of 100 kD was used to remove endotoxin. After ultrafiltration, the content of endotoxin in phage preparation was less than 100 EU/ml. Then the phage concentration was adjusted to 10^12^ PFU/ml, and the formaldehyde was added to phage particles at a volume ratio of 1:4,000 to inactivate the phage to obtain the GAD65 phage vaccine.

### Animal Immunization

Female NOD mice of 4–6 weeks of age were purchased from GemPharmatech Co, Ltd (Nanjing, China). All the animals were housed in a light- and temperature-controlled environment. All animal studies (including the mice euthanasia procedure) were done in compliance with the regulations and guidelines of the Institute of Medical Biology, Chinese Academy institutional animal care and conducted according to the AAALAC and the IACUC guidelines.

**Figure 1:**
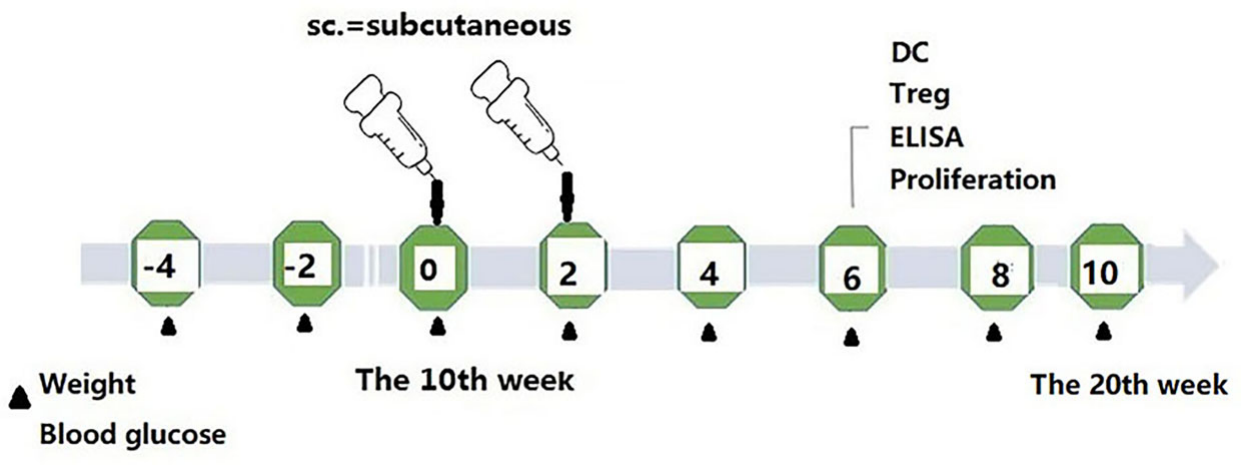
Schematic Map of immunization schedule.

The mice were randomly divided into four groups (12 animals per group). They were subcutaneously immunized (subcutaneous injection at different sites on the back of mice) with phage vaccine alone or co-immunized with Kyn (Sigma, K8625) in 100 μl final volume listed in **Table 1**. The animal immunization schedule and detection program are shown in **Figure 1**. Briefly, mice were sacrificed, and splenocytes were harvested at different time points. Blood samples were collected, and sera were stored at −20°C until tested.

**Table 1:** Animal groups and dosage.

Groups	Dose	Adjuvant	Injection Times	No.
Control	100 μl (10^12^Pfu/ml)		2	12
Control + KYN	100 μl (10^12^Pfu/ml)	200 μg KYN	2	12
GAD65	100 μl (10^12^Pfu/ml)		2	12
GAD65 + KYN	100 μl (10^12^Pfu/ml)	200 μg KYN	2	12

### Antibodies and Synthetic Peptide

Fluorescently labeled anti-mouse monoclonal antibodies including anti-mouse CD4-FITC (RM4-5), anti-mouse CD25-APC (PC61.5), anti-mouse/Rat-Foxp3 PE (FJK-16s), anti-CD11c-PE (N418), anti-CD80-APC (16-10A1), anti-IL10-FITC (JESS-16E3), and isotype controls were purchased from eBioscience (San Diego, CA, USA). GAD65 specific peptide acid sequences were TYEIAPVFVLLEYVT, EYVTLKKMREIIGWPGGSGD, KKGAAALGIGTDSVI, ALGIGTDSVILIKCDERGK.

### Determination of Serum Antibodies and Cytokines

#### Determination of Serum Antibodies by ELISA

The 96-well flat-bottom plates were coated with purified recombinant GAD65 protein (purified by our lab) at a concentration of 0.1μg/ml in coating buffer at 4°C overnight. Plates were washed five times with washing buffer and blocked with blocking solution at 37°C for 1 h. After that, mice sera were serially diluted two-fold in blocking solution (starting at 1:100–1:3,200), and 100μl was added to each well. After incubation for 1 h, the plates were washed five times and incubated with 1:2,000 diluted HRP-labeled goat anti-mouse IgG1 and IgG2a antibodies (Invitrogen, USA) at 37°C for 1 h. After the final wash, 100μl of ABTS HRP substrate was added per well and plates were incubated for 5 min; the reaction was stopped by adding 25 μl of ABTS peroxidase stop solution. The optical density (OD) of the plate was measured at 450 nm by ELISA plate reader (UVP, California, USA). The ELISA test reagents were from the ELISA Kit Anti-Mouse ABTS^®^ System (KPL Protein Detector™).

#### Determination of Serum Antibodies and Cytokines by Electrochemiluminescence

The concentrations of the IL-2, IL-4, IL-10, IFN-*γ* and TgF-*β*_1_ in the serum samples were examined using the MSD ECLIA according to the manufacturer’s instructions (MSD, Rockville, MD, USA). For quality control, a standard curve was prepared. The highest point and background point were selected for the standard point to confirm that the sample concentration was within the range of the standard curve. In the preliminary experiment, the sample was diluted two and eight times; eight times had undetectable factors, which indicated that dilution of at least two times was recommended. Three repeated tests were performed on each sample.

### Spleen Lymphocyte Proliferation Analysis

Mice splenocytes were harvested after 4 weeks of the last treatment. Splenocytes were recovered as a single cell suspension in RPMI 1640 medium (Hyclone). Erythrocytes were removed from splenic suspensions by lysis in ACK Lysis Buffer (Gibco™) for 2 min at 37°C. After that, the 1,640 complete culture medium containing 10% fetal bovine serum was added, and the cells were inoculated into 96-well plates at the concentration of 1 × 10^5^/ml. GAD65 specific peptides were added as antigen stimulation (final concentration 5 μg/ml); PMA (positive control, final concentration 5 μg/ml) was added as positive antigen control. After 48 h of stimulation, the 20 μl MTS (Promega) was added to each well at the last 4 h of incubation. The wavelength of 490 nm was used to measure light absorption value of each well by ELISA plate reader (UVP, California, USA).

### Flow Cytometry Assay

#### Analysis of DC

Mice splenocytes were harvested after 4 weeks of the last treatment. Mice spleen lymphocytes were harvested and washed two times with PBS containing 2% BSA. Cells were then adjusted to the concentration of 1 × 10^6^/ml and incubated for 48 h at 37°C and 5% CO_2_ with 100 μl GAD65 peptides pool containing 5 μg/ml of each individual peptide as described previously. Cells were processed as described previously and stained with anti-CD11c-PE (N418) and anti-CD80-APC (16-10A1). After permeabilization, samples were stained with intracellular anti-IL10-FITC (JESS-16E3) fluorescent-labeled monoclonal antibody. After washing two times, samples were re-suspended with PBS and immediately analyzed on CytoFLEX S Flow Cytometer (BECKMAN COULTER, USA). The flow data was processed using FlowJo10.4 software.

#### Analysis of Treg

Mice spleen lymphocytes were harvested as previously described, and then adjusted to the concentration of 1 × 10^6^/ml and incubated for 48 h at 37°C and 5% CO_2_ with 100 μl GAD65 peptide pool containing 5 μg/ml of each individual peptide as described previously. After that, cells were stained with anti-mouse CD4-FITC (RM4-5), anti-mouse CD25-APC (PC61.5) antibodies. Then, the cells were fixed with Fix/Perm buffer (eBioscience, America), incubated in permeabilization buffer (eBioscience, America), and stained with anti-mouse/Rat-Foxp3 PE (FJK-16s) antibody. After washing two times, samples were re-suspended with PBS and immediately analyzed on CytoFLEX S Flow Cytometer (BECKMAN COULTER, USA) as previously described.

### RNA Sequencing

We also provided insight into the mechanisms of Kyn and its influence on the immune response to immune cells. Mouse splenocytes were performed to assess the immune suppressive properties of kynurenine and to determine that the adjuvants used were indeed biologically active. Balb/C mice spleen lymphocytes (three mice in each group) were isolated as described above, after that they were stimulated for 12 h by Kyn (40 μg/ml), which performed at least three independent experiments. Splenocyte samples were collected after treatment, after which the total mRNA and microRNA libraries were prepared and sequenced. Total RNA was extracted using the RNAfast200 kit (Fastagen Biotech, Hefei, China) according to the manufacturer’s protocol. The quality control of the isolated RNA (concentration, RIN, 28S/18S, and size) was performed with Agilent 4200 Bioanalyzer (Agilent Technologies, Santa Clara, USA). The strand-specific RNA-seq libraries were prepared using a NEBNext^®^ Ultra™ I RNA Library Prep Kit for Illumina (NEB, MA, USA) following the manufacturer’s instructions. The libraries were assessed on the Agilent Bioanalyzer 4200 system and sequenced on the Illumina Xten platform. RNA sequencing and reads alignment were performed by GMINIX Biotechnology Corporation (Shanghai, China). Reads were aligned to mouse genome version mm9. MicroRNA sequencing and RNA sequencing data are deposited at the Gene Expression Omnibus (GEO; accession number: GSE164304; GSE165737). Differentially expressed miRNAs/mRNAs were selected based on the following criteria: |log2 fold change| > = 1.2 and P-value <0.05.

### Validation of Differentially Expressed miRNA and mRNA

The expression of the selected miRNAs was detected by the stem-loop qRT-PCR method. Total RNA was extracted to obtain cDNA by reverse transcription-PCR using GoScript Reverse Transcription System (Promega, USA) according to the manufacturer’s protocol. The qPCR assay was performed in CFX96 Touch Real-Time PCR Detection System (BioRad, Berkeley, USA). The cycling parameters of qPCR reaction were as follows: 95°C for 5 min, then 95°C 10 s, and 60°C 30 s for 40 cycles (miRNAs); 95°C for 5 min, then 95°C 10 s, and 50°C 30 s for 35 cycles (mRNAs), followed by a melting curve to record the specific PCR product. In the qPCR experiment, the relative expressions were calculated using the 2–ΔΔCt method with GAPDH as an endogenous control for mRNA and U6 as an internal control for miRNA. Each reaction was conducted in triplicate. The corresponding primers were shown in **Supplementary Table 1**.

### Statistical Analyses

Data are presented as means ± standard deviations (SDs), and statistical analyses were performed with professional statistical computer SPSS software. A p value of <0.05 was considered statistically significant.

## Results

### The Effect of Kyn on Suppressed Hyperglycemia and Diabetes

**Figure 2:**
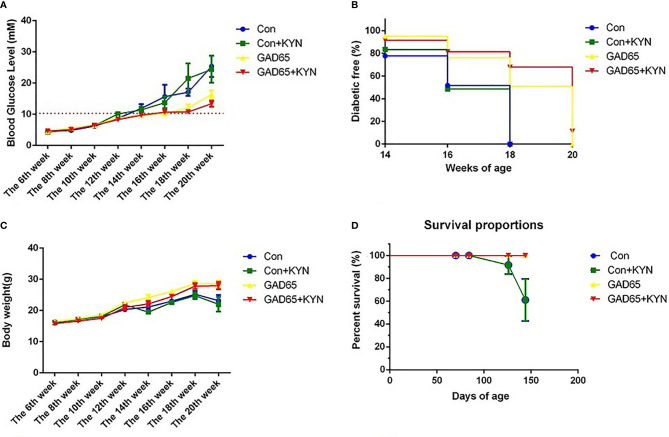
NOD mice were immunized with phage vaccine with or without KYN on weeks 10 and 12. **(A, C)** Blood glucose/body weight was measured on weeks 6, 8, 10, 12, 14, 16, 18, and 20. The body weight of mice grew when treated with GAD65 phage vaccine with KYN. **(B)** Mice immunization of GAD65 phage vaccine with KYN were significantly remission from hyperglycemia in 60% at least one month. **(D)** The survival curves.

To study the potential immunosuppressive adjuvant effects of Kyn, we immunized mice with the GAD65 phage vaccine premixed with the Kyn adjuvant. The immunization and sample collection schedule is shown in **Figure 1**. Blood glucose levels and body weight of NOD mice were detected at 6, 8, 10, 12, 14, 16, 18, and 20 weeks. Our results showed that (**Figures 2A, B** **)** subcutaneous administration of GAD65 vaccine, or GAD65 vaccine + Kyn, prevented the development of hyperglycemia in 50% (3/6) and 67% (4/6) of NOD mice at least one-month (from the 14th week to 18th week), respectively. Clinical diabetes was defined by hyperglycemia (blood glucose levels >10.3 mmol/l) in fasted animals (27); the protective effect began after the 14^th^ week of initial hyperglycemia in our study. In contrast, no significant remission of hyperglycemia was observed in the control group and control + Kyn group. Weight loss is a significant symptom of T1D, and we found no changes in weight loss in GAD65 and GAD65 + Kyn groups. During the 144 days of observation period (**Figures 2C, D** **)**, mice in control and control + Kyn groups had a mortality rate of 33.3% (2/6), while no death was observed in GAD65 and GAD65 + Kyn groups (mice were in good condition, weighing between 25 and 30 g). Thus, these results indicated that co-immunization of Kyn and GAD65 phage vaccine could significantly temporarily reverse diabetes in NOD mouse model.

### The Effect of Kyn Shifted the Th1/Th2 Balance Toward Th2

**Figure 3:**
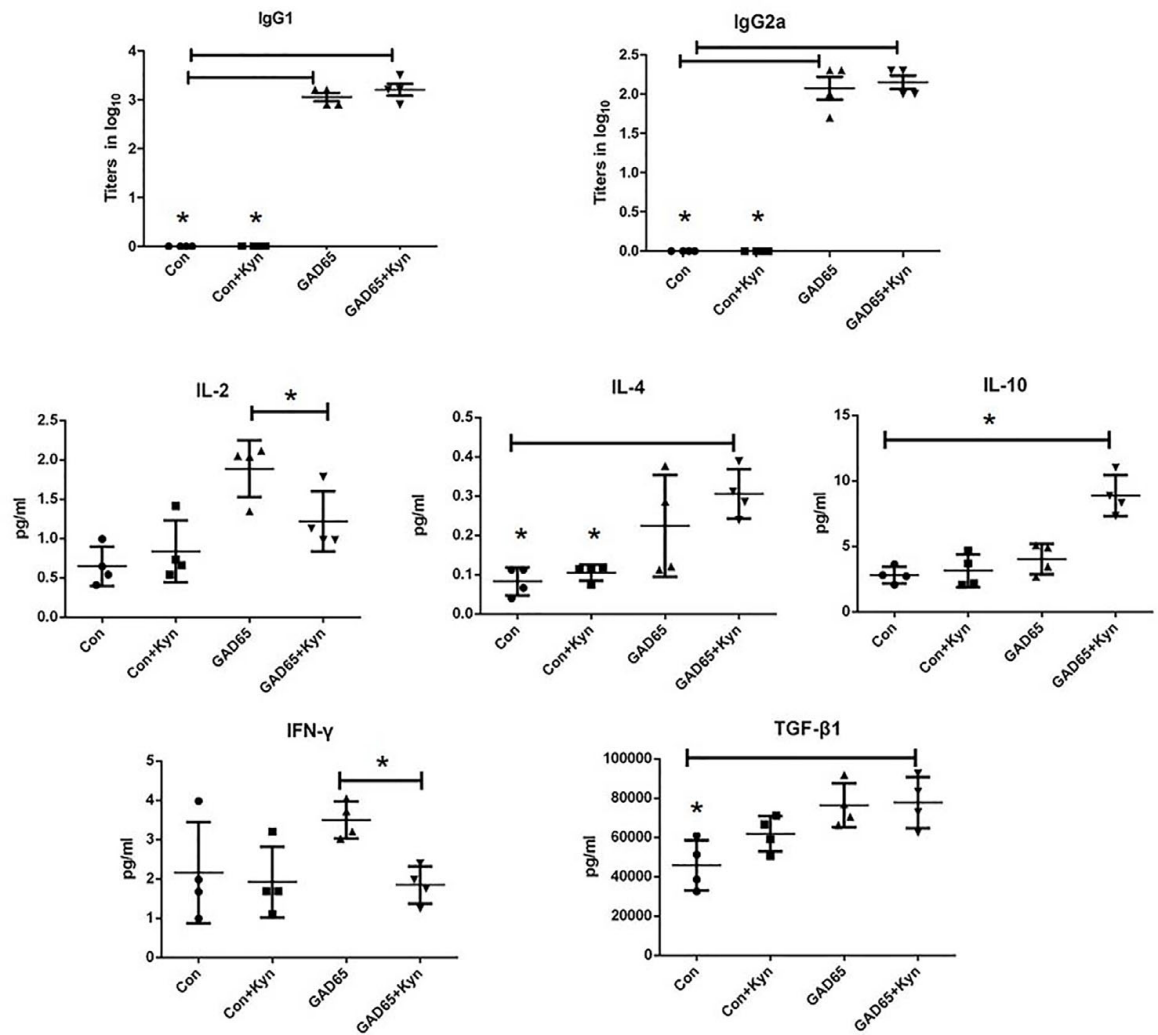
Sera samples were collected to detect cytokines with MSD electochemiluminescence. GAD65 phage vaccine co-immunized with KYN generated significantly lower secretion of IL-2, IFN-*γ* than the GAD65 phage group did, and higher secretion of IL-10, IL-4, TGF-*β*1, than any other groups did (*P < 0.05).

Since the proportion of Th1/Th2 cells is not balanced and the immune response is skewed to Th1 in diabetic NOD mice and diabetic patients, it is important to examine whether Kyn can decrease GAD65-specific inflammatory profiles of T cells. Comparing the antibody responses generated by GAD65 phage immunizations with or without Kyn adjuvant, the IgG1 and IgG2a isotypes produced by both immunization ways were detected. DNA injection into the muscle can produce a Th1 type immune response, and mainly IgG2a antibodies in mouse models (28). Compared to the group immunized with GAD65 phage alone, a significantly enhanced level of IgG1 was obtained in the group immunized with GAD65 phage plus Kyn as an adjuvant. IgG1 anti-GAD65 antibodies resulted in ratios of IgG1 to IgG2a >1 that was indicative of Th2 polarization (**Figure 3**).

To further determine whether the immunosuppressive effect of Kyn on diabetes was mediated by Th2 cells, electrochemiluminescence assay was performed to examine the effects of Kyn on the generation of Th1 and Th2 cytokines. We detected the production of IFN-*γ*, IL2, IL-4, IL-10 cytokines associated with Th1 and Th2 responses in the serum of immunized NOD mice. Our results revealed that IL-4 and IL-10 (Th2 cytokines) were significantly up-regulated (**Figure 3**), while production of IL-2 and IFN-*γ* (Th1 cytokines) was significantly down-regulated in Kyn co-immunized mice with GAD65 phage vaccine compared with other groups, which is consistent with IgG isotype data. These results indicated that Kyn could enhance the GAD65 vaccination by shifting the Th1/Th2 balance toward Th2. We also found that the Tgf*β*1 was increased when immunized with the adjuvant Kyn + GAD65 vaccine compared with other groups in NOD mice.

### GAD65 Phage Vaccine + Kyn Decreases GAD65-Specific T Cell Proliferation

**Figure 4:**
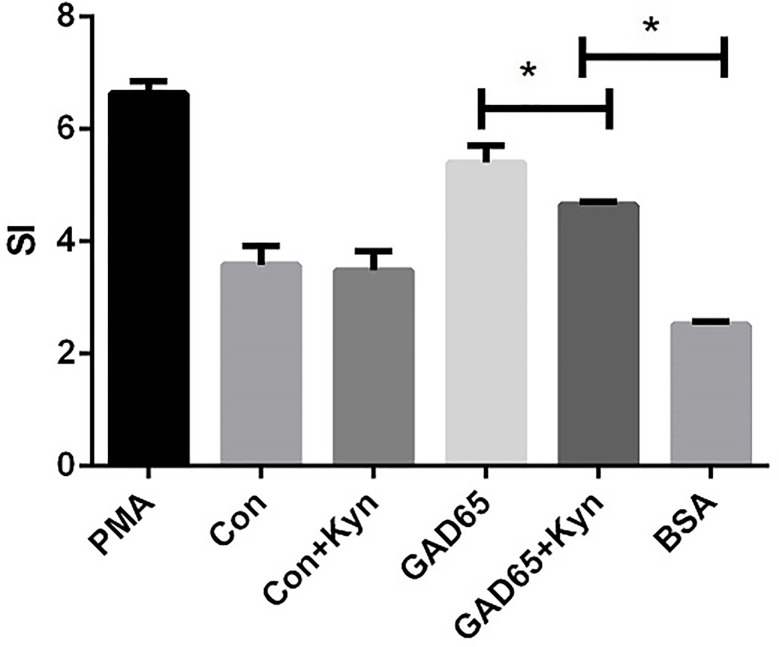
Effects of suppressive adjuvant Kyn on the GAD65 specific T cell proliferation. The proliferative response to GAD65 was significantly lower in spleen lymphocytes isolated from GAD65 phage vaccine + Kyn immunized mice than those from the GAD65 phage vaccine immunized mice (*P < 0.05).

In the case of T1D, auto-reactive T cells attack islet cells, most of which are insulin-specific cytotoxic CD8^+^ T cells. To explore the potential underlying mechanisms of GAD65 phage vaccine + Kyn on diabetes, we measured the autoantigen-specific T cell activation and proliferation *in vivo* using T cell proliferation assay in response to GAD65. As shown in **Figure 4**, the proliferative response to GAD65 was significantly lower (P < 0.05) in spleen lymphocytes isolated from GAD65 phage vaccine + Kyn immunized mice than those from the GAD65 phage vaccine immunized mice. The proliferation was specific to GAD65 peptide since challenge with the control BSA peptide *in vitro* had no effect (P < 0.05). Our data demonstrated that GAD65 phage vaccine + Kyn vaccination could inhibit the proliferation of GAD65 auto-reactive T cells *in vivo*.

### Kyn Can Suppress Dendritic Cell Maturation

**Figure 5:**
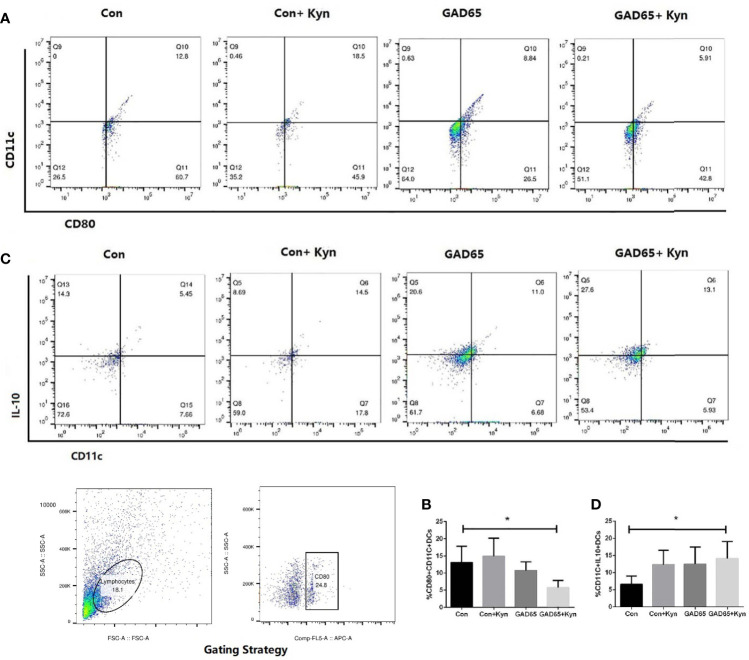
Kyn suppressed mouse dendritic cell maturation. At the 16th week, mice spleen lymphocytes were stained and immediately analyzed on Flow Cytometer. Compared with GAD65 phage vaccine immunization alone, significantly lower percentages of CD80^+^CD11c^+^ DC cells were observed in GAD65 phage vaccine co-immunized with KYN, which suggested that KYN may suppress dendritic cell maturation **(A, B)**. At the same time, immatured-dendritic cells in the co-immunized group secreted more IL10 than the control group did **(C, D)** (*P < 0.05).

DC promotes immunity and mediates T cell tolerance by direct elimination, Treg induction, or co-adjustment. Immune tolerance can be induced by adoptive transfer of immature or semi-mature DCs, or by self-antigen-presenting DCs under steady-state conditions (29). Twenty-eight days after the last immunization, single cells of the spleen lymphocyte were collected, stained with anti-CD11c-FITC, anti-CD80-APC, and anti-IL10-PE, and analyzed by fluorescence-activated cell sorting (FACS) to detect the mature state of DCs. As shown in **Figures 5A, B**, the proportion of mature DC cells (CD11c^+^CD80^+^) in the group immunized by Kyn + GAD65 was significantly lower than that in the group immunized by GAD65 alone, indicating that Kyn can inhibit the maturation of DC cells when stimulated by GAD65 peptides (P < 0.05).

We also detected the changes in IL-10 expression in DC cells (**Figures 5C, D** **)**. The highest percentage of IL-10 expression in DC cells immunized by Kyn + GAD65 phage vaccine was 14.2%, which was significantly higher compared to other groups, indicating that Kyn could enhance the ability of DCs to express IL-10. At the same time, we also observed that Con + Kyn or GAD65 phage vaccine group could induce more secretion of IL10 in immature DC cells compared with the control group (P < 0.05). This result confirmed that GAD65, as an autoimmune antigen of T1D, can induce certain immune tolerance, and Kyn could enhance this tolerance effect.

### GAD65 Phage Vaccine + Kyn Increases CD4^+^CD25^+^ Treg Cells in the NOD Mouse Model

**Figure 6:**
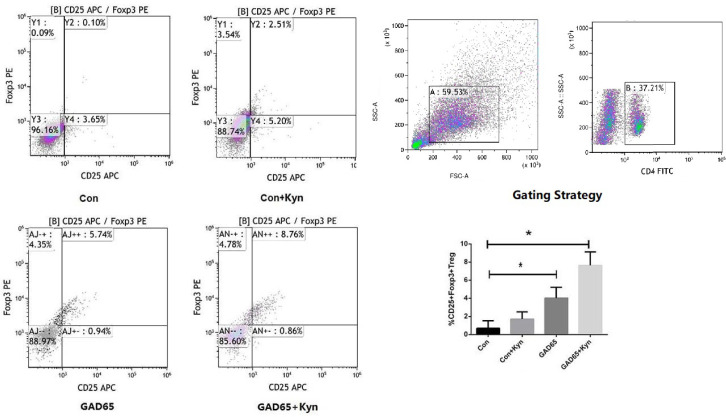
Kyn induced CD4^+^CD25^+^Foxp3^+^ Treg cells. At the 16th week, mice spleen lymphocytes were stained and immediately analyzed on Flow Cytometer. Notably, Kyn still strongly enhanced the percentages of CD4^+^CD25^+^Foxp3^+^ Treg cells when co-immunized with GAD65 phage vaccine (*P < 0.05).

Treg is a subset of CD4^+^ T cells which are characterized by the expression of transcription factor Foxp3 and helps to keep inflammation under control and lower the autoimmune disease risk in healthy individuals (30). They have been proved to maintain their regulatory functions for a long-term even in the absence of antigens that induced their generation and are stable and transferable (31), thereby permitting the expected induction of these cells to suppress unwanted immunity (13). In this study, we evaluated the effect of the Kyn co-immunized with GAD65 phage vaccine on the regulation of Treg cells in spleen lymphocytes. Spleen lymphocytes were isolated and re-stimulated in culture with the GAD65 peptides. The cells were then stained with anti-CD4-FITC, anti-CD25-APC, anti-FoxP3-PE and analyzed by fluorescence-activated cell sorting (FACS). As shown in **Figure 6**, with the administration of Kyn, the Kyn + GAD65 group produced a higher proportion of CD4^+^CD25^+^Foxp3^+^ T cells in CD4^+^ T cell populations compared with the GAD65 group (P < 0.05). A similar result was observed in the GAD65 group; the proportion of CD4^+^CD25^+^Foxp3^+^ T cells in CD4^+^ T cell populations was significantly improved compared with control or control + Kyn group, respectively (P < 0.05). These results indicated that GAD65, as an autoimmune antigen of T1D, could increase the proportions of CD4^+^CD25^+^Foxp3^+^ T cells, and immunosuppressive adjuvant Kyn could specifically increase this enhanced effect, which may be associated with the suppression of T1D progression in the spleen lymphocytes.

### Gene Expression Profile of Spleen Lymphocytes Stimulated by Kyn *In Vitro*

#### Analysis of miRNA Expression Profiles

RNA-sequencing technology uses ultra sequencing technologies to determine vaccine adjuvant transcriptomic profiles *in vivo* and *in vitro* (32–34 *).* Modigliani Y et al., confirmed that miRNA expression has an important role in vaccination with aluminum adjuvant (32). In our study, 91 distinctly expressed miRNAs were identified between the Kyn stimulated group and the NC group (>1.2-fold change and p < 0.05 as screening criteria). Among them, 46 (50.5%) miRNAs were up-regulated, and 45 (49.5%) were down-regulated (**Supplementary Figure 2A**). The detailed information of differentially expressed miRNAs can be seen in **Supplementary Table 2**. Additionally, hierarchical clustering analysis showed the expression profile of 91 differentially expressed miRNAs in different treatment groups. The results indicated that these miRNAs were divided into two major clusters, one representing the negative controls and one representing Kyn stimulated group. The data illustrated that these common expression patterns of miRNAs were significantly different from NC groups (**Supplementary Figure 2B**). Validation of expression levels for randomly selected miRNAs was analyzed using qRT-PCR (**Supplementary Figure 1A**); the results were consistent with the expression trends shown in the miRNA sequencing results for the selected miRNAs.

#### Analysis of mRNA Expression Profiles

Transcriptome sequencing was used to analyze the mRNA expression profiles of spleen lymphocytes stimulated by Kyn (>1.2-fold change and p<0.05 as screening criteria). After 12 h of stimulation, differentially expressed mRNAs matching these criteria were depicted in volcano maps (**Supplementary Figure 2C**). A total of 1,436 differentially expressed genes were significantly different between the Kyn stimulated group and the NC group (**Supplementary Table 3**). Among these differentially expressed mRNAs, 713(49.7%) were up-regulated and 723 (50.3%) were down-regulated. Additionally, cluster analysis indicated that the expression of differentially expressed mRNAs was significantly different between the Kyn-stimulated group and the NC group (**Supplementary Figure 2D** **)**. Validation of these trends was performed on randomly selected mRNAs and was demonstrated using qRT-PCR (**Supplementary Figure 1B**).

#### miRNA Target Prediction and Integration of miRNA and mRNA Expression Profiles in Kyn-Stimulated Spleen Lymphocytes

##### Functional Analysis of Common Differentially Expressed miRNAs

**Figure 7:**
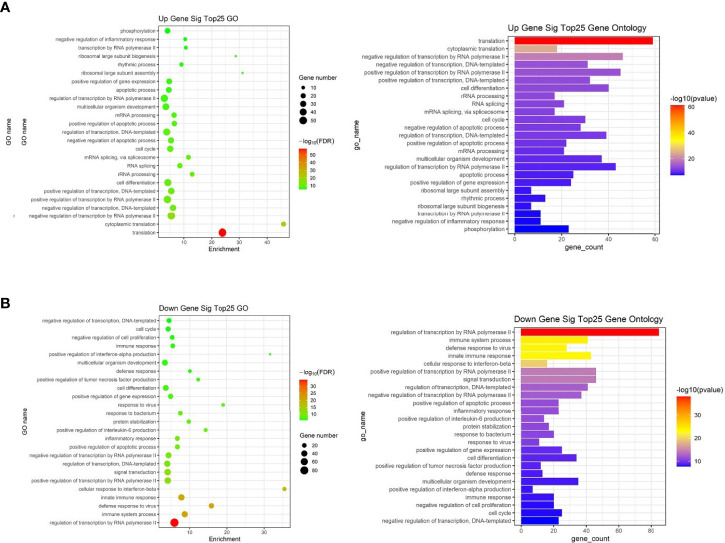
The top 25 significantly enriched GO terms of the overlapping target genes of differentially expressed miRNAs in spleen lymphocytes of Balb/C mice (Kyn stimulated *vs* negative control). **(A)** Up gene Go analysis. **(B)** Down gene Go analysis.

To further study the role of the identified differentially expressed miRNAs, we first predicted the miRNA target genes *via* targetscan (http://www.targetscan.org/, mirnada (http://www.microrna.org/and miRWalk (http://129.206.7.150/) databases. A total of 1,073 target mRNAs were predicted from the intersection of the three databases. These target mRNAs, which were predicted by the bioinformatics software and confirmed by transcriptome sequencing, were identified as the preliminarily putative target genes. Then, the preliminarily putative target genes that present a negative regulatory relationship between miRNA-target mRNAs were included. Finally, the differentially expressed target mRNAs that passed through these screen processes were subject to analysis using Gene Ontology (GO) and Kyoto Encyclopedia of Genes and Genomes (KEGG). All Gene Ontology can be seen in **Supplementary Table 4**, the significant top 25 GO terms were described in **Figure 7**. Up gene GO analysis (**Figure 7A**) results showed that the five most-enriched terms are translation, cytoplasmic translation, negative regulation of transcription by RNA polymerase II, negative regulation of transcription, DNA-templated and positive regulation of transcription by RNA polymerase II. Down gene GO analysis (**Figure 7B**) results showed that the five most-enriched terms are regulation of transcription by RNA polymerase II, immune system process, defense response to virus, innate immune response and cellular response to interferon-beta. These data could provide evidence that miRNA potentially up-regulated GO is mostly involved in gene transcription, but down-regulated GO is mostly involved in immune response.

**Figure 8:**
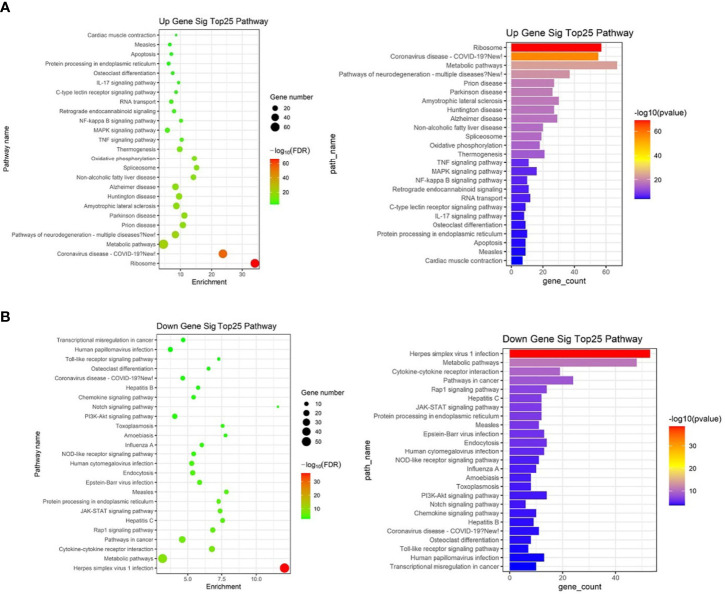
The top 25 significantly enriched KEGG-pathway analyses of overlapped target genes of differentially expressed miRNAs in spleen lymphocytes of Balb/C mice (Kyn stimulated *vs* negative control). **(A)** Up gene KEGG analysis. **(B)** Down gene KEGG analysis.

**Figure 9:**
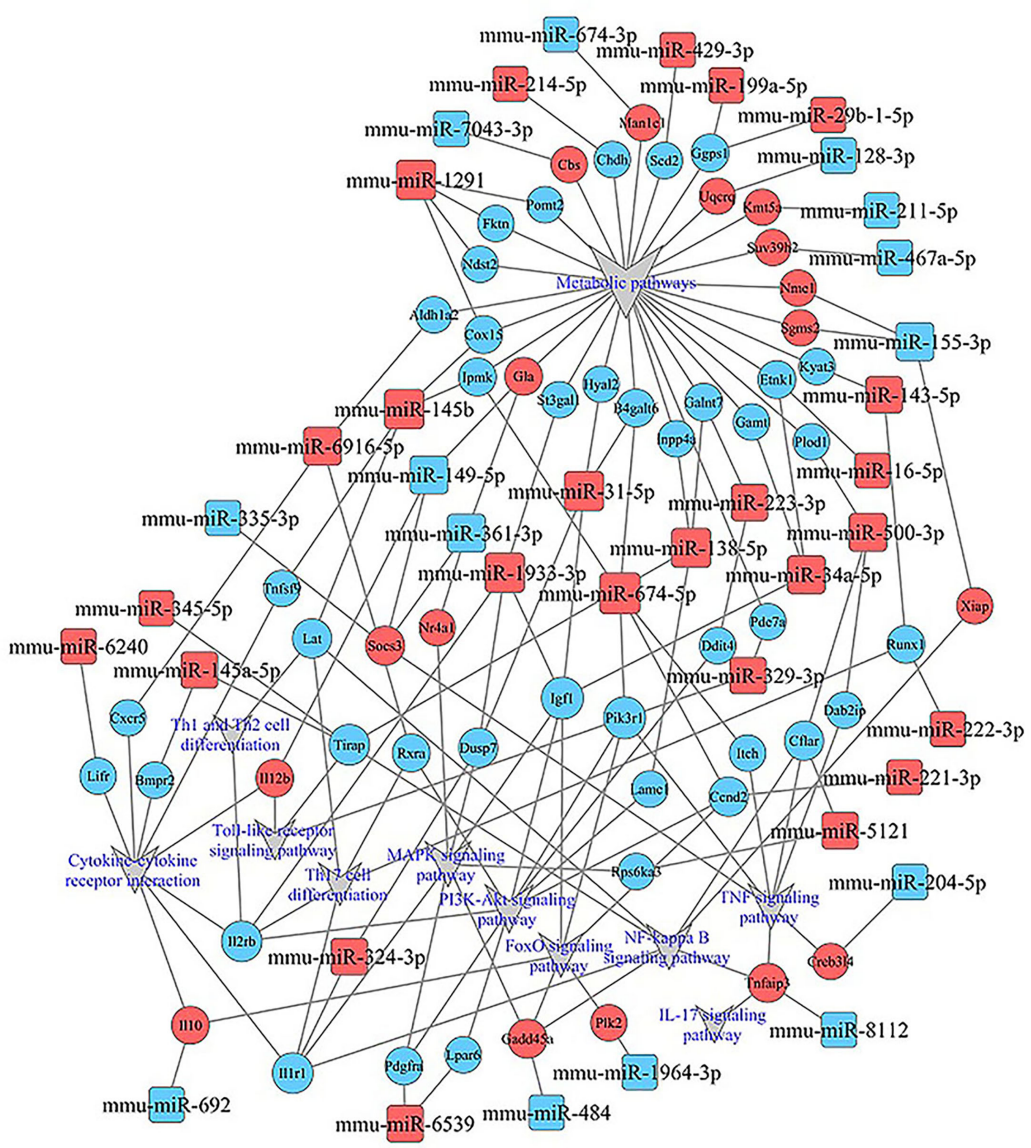
miRNA–mRNA-pathway-net. Squares indicate identified miRNAs, while circles represent the corresponding target genes. Blue indicates down-regulated miRNAs or mRNAs while red indicates up-regulated miRNAs or mRNAs. The relationship between miRNAs and genes is shown connected by gray lines. Eleven significantly enriched pathways associated with splenocytes stimulated by Kyn were depicted using the gray triangles. More details about immune related miRNA–mRNA-pathway are shown in **Supplementary Figure 3**.

KEGG analysis of these target mRNAs illustrated that the putative target genes of the selected common miRNAs were enriched into 186 KEGG signaling pathways. Of these signaling pathways, 91 KEGG signaling pathways were up-regulated, and 95 were down-regulated. The top 25 signal pathways with the most enriched genes were depicted by bubbles (**Figure 8**). Among those signaling pathways **(** **Supplementary Table 5** **)**, all involved in the modulation of Kyn stimulated signal pathways, including metabolic pathways, FoxO signaling pathway, cytokine–cytokine receptor interaction, Toll-like receptor signaling pathway, NF-kappa B signaling pathway, TNF signaling pathway, MAPK signaling pathway, IL-17 signaling pathway, were enriched in both up and down pathways. Th17 cell differentiation, Th1 and Th2 cell differentiation, PI3K–Akt signaling pathways were significantly enriched in down pathways. In general, these pathways mostly focused on metabolic regulation and immune regulation. These 11 pathways were selected, and the miRNA–mRNA–pathway regulatory network was generated based on the previously selected miRNA-target mRNAs pairs (**Figure 9**). There were 39 differentially expressed miRNAs including 25 up-regulated miRNAs and 14 down-regulated miRNAs, and 58 differentially expressed mRNAs included 17 up-regulated mRNAs and 41 down-regulated mRNAs which were involved in these 11 pathways. In the miRNA–mRNA-pathway-net, we observed that some genes have an important regulatory effect in these pathways that up-regulated gene Gadd45a, Il12b, IL10 and down-regulated gene Igf1, Il1r1, PIK3R1 which were located in the center nodes (**Supplementary Figure 3**).

##### Functional Analysis of Specific Differentially Expressed miRNAs

**Figure 10:**
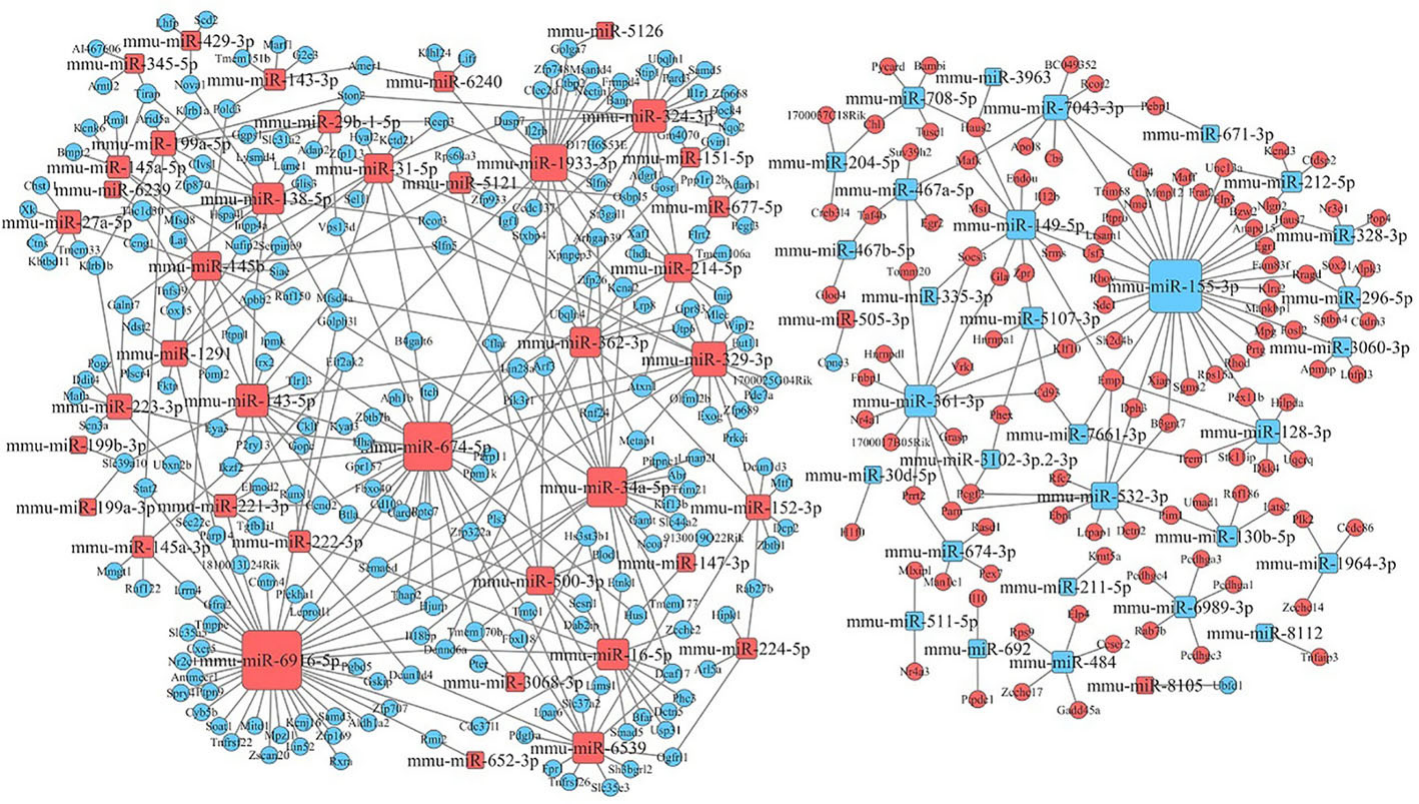
miRNA–mRNA interaction network. The size of the point represents the regulatory capacity of a given miRNA. Squares indicate identified miRNAs, while circles represent the corresponding target genes. Blue indicates down-regulated miRNAs or mRNAs while red indicates up-regulated miRNAs or mRNAs. The relationship between miRNAs and genes is shown connected by gray lines.

After KEGG signaling pathway enrichment analysis, the miRNA–mRNA regulatory network map was generated in **Figure 10**. Bioinformatics analysis demonstrated that some specific miRNAs play a significant role in regulating the lymphocyte function when stimulated by Kyn. Among the up-regulated miRNAs with more predicted targets, mmu-miR6916-5p had 40 predicted targets, followed by mmu-miR674-5p and mmu-miR34a-5p with 33 and 22 predicted targets, respectively. There were few predictive targets for down-regulated miRNAs, although mmu-miR155-3p had 34 predictive targets. Consequently, our data could provide evidence that there is a close relationship between miRNA changes, host gene expression, and suppressive adjuvant effects.

## Discussion

Although animal model studies have demonstrated that GAD65 vaccination can be used to prevent autoimmune diabetes (35–39), the preventive efficacy needs to be further improved. Successful clinical treatment may be hindered by the uncertainty of major auto-antigens, an insufficient dosage of antigen and lack of an ideal adjuvant or low Treg induction to confer long time tolerance effects (40). In order to address these issues, we designed an immunosuppressant-kynurenine (Kyn), as adjuvant co-immunized with a phage-displayed vaccine containing the 190–320 amino acid sequences of GAD65 to induce Treg cells and tolerogenic responses and to prevent diabetes I disease in the NOD mouse model.

Our study indicated that subcutaneous administration of GAD65 phage vaccine + Kyn could prevent the development of hyperglycemia in 60% (4/6) of NOD mice for at least one month, which was highly effective for the suppression of T1D in NOD mice. Weight loss is a significant symptom of T1D. In our study, no significant changes in weight loss were observed in the GAD65 phage vaccine and GAD65 phage vaccine + Kyn groups. In addition, no death was observed in these two groups, and mice were in good condition (weighing between 25 and 30 g). This greater efficacy may be due to the combination of GAD65 antigen with the immunosuppressant-kynurenine, thus providing effective immunotherapy against T1D.

In T1D, GAD65 has been identified as a primary auto-antigen in the pancreas. Some studies (41–46) indicated that the administration of recombinant GAD65 protein or peptide in NOD mice can induce immune tolerance against pancreatic *β* cells and thus prevents or delays the development of insulitis and diabetes. Based on human clinical trials and animal studies (47), the proposed mechanism includes induction and proliferation of GAD65-specific Tregs, which down-regulate antigen-specific auto-reactive T cells and prevent them from attacking the pancreatic *β*-cells. Therefore, inducing the proliferation of antigen-specific regulatory T cells may be one of the most effective ways to prevent or treat autoimmune diseases.

The use of Tregs as a method to treat inflammation and autoimmune diseases has been proposed and well-received by scientists (30). Many clinical trials have proved that the use of vaccines to treat autoimmune diseases cannot effectively induce the production of antigen-specific Treg cells, thus inhibiting the role of pathological T cells. Consequently, it is necessary to add inhibitory adjuvants in the process of vaccine use. Our previous studies have shown that Kyn may be used as a suppressive adjuvant to reduce the immunogenicity of HAV, a TD antigen, *in vivo*, and LPS, a TI antigen, *in vitro* (48 *).* As an endogenous ligand of AHR, Kyn can activate the AHR signaling pathway and may control immunity and autoimmunity while providing us with a new opportunity for autoimmune disease therapeutic. In this study, GAD65-specific CD4^+^CD25^+^FoxP3^+^Treg cells were significantly increased in the GAD65 vaccine + Kyn group compared with the vaccine single immunization group.

We also discovered that the proportion of mature DC cells (CD11c^+^CD80^+^) was significantly lower in mice co-immunized with Kyn, indicating that Kyn as an adjuvant could inhibit the maturation of DC cells. The highest proportion of IL-10 expression in DC cells in the GAD65 co-immunization Kyn group was 14.2%, which was significantly higher than that in other groups, indicating that Kyn could enhance the ability of DC cells to express IL-10. The mature state of DC cells stimulated by antigen directly affected the production of the immune response. Under normal physiological conditions, the fully matured DCs secrete the pro-inflammatory cytokines, such as IL-1β, TNF, IL-12, and IL-6 (49). However, DCs can also perform the opposite function by making T cells tolerant against the autoantigen-directed immune response, which is necessary to reduce the autoimmune reactions. The immunogenic and tolerogenic functions of DCs depend on the balance between activating and inhibitory signals during DC maturation (50).

In T1D patients and NOD animal models, the skewed Th1/Th2 balance which leads to autoimmune destruction of the *β*-cells in the pancreas is a progressive phenomenon, resulting in the continual loss of these cells (51, 52). Our results showed higher IgG1 than IgG2a in both GAD65 vaccine and GAD65 vaccine + Kyn adjuvant groups, indicating that the vaccine treatment could induce Th2 type immune response in NOD mice, mainly regulating humoral immunity. In our study, protection from diabetes by Kyn + GAD65 phage immunization was also associated with a significant reduction of T cell proliferation and of IFN-*γ* secretion by T cells responding to GAD65. Additionally, an increase in the release of IL-10 and TGF*β*1 was detected in Kyn + GAD65 phage vaccinated mice, indicating that Kyn + GAD65 phage immunization shifted the diabetogenic Th1 response to the regulatory Th2 response. IL-10 is a strong anti-inflammatory cytokine, which plays an important role in inhibiting Th1 cells by inhibiting the production of proinflammatory cytokines such as IFN-*γ*, IL-1 and TNFα. TGF-*β*, which induces immune tolerance and counteracts the immunostimulatory effects of checkpoint inhibitors, has an immunosuppressive effect (53). The secretion of IL10 and TGF-*β* and the production of Treg cells complement each other, which is the key factor for the feasible treatment of autoimmune diseases. T1D is related to the immune imbalance caused by excessive activation of Th1 cells and inhibition of Th2 cells. Therefore, whether T1D can be cured or not depends on whether the damaged Th1/Th2 balance can be effectively restored by immune regulation.

Our data suggested that Kyn can be used as an immunosuppressive adjuvant in autoimmune disease. Understanding how lymphocytes interact with adjuvants is crucial to understanding the mechanisms of this adjuvant and will be critical in the rational design of future vaccines against many diseases. In the present study, RNA and miRNA sequencing was performed in mouse spleen lymphocytes stimulated by Kyn. Compared with the negative control group, 91 common differentially expressed miRNAs and 1,436 common differentially expressed mRNAs were found in the Kyn stimulated group. The results of the GO analysis revealed that the up-regulated genes were mainly enriched in gene expression processes, such as mRNA processing, RNA splicing and translation. The down-regulated genes were primarily involved in immune-related biological processes, such as positive regulation of tumor necrosis factor production, interferon-alpha production, and IL-6 production. According to the KEGG‐pathway analysis, both up-regulated and down-regulated genes play a crucial role in metabolic pathways and pathways related to immune regulatory function. This is in agreement with other studies in which a relationship between metabolic state and the differentiation status of innate and lymphoid cells (54).

Numerous studies (55) have demonstrated that AhR plays an important role in several normal physiological processes, including development of the vasculature, construction of the central nervous system, differentiation of blood cell subsets, and the maintenance function of hepatocytes, adipocytes, and epithelial cells. Beyond that, the AhR plays a crucial role in the control of the adaptive immune response. It controls the differentiation and activity of specific T-cell subsets and influences adaptive immune responses by affecting both T cells and antigen presenting cells (APCs). Kyn is the first metabolite of tryptophan. As an endogenous substance, it activates the AHR in a ligand-receptor manner. In our study we found that Kyn can work as immunosuppressive adjuvant and mainly has a negative regulatory role in the immune related signaling pathway, offering plausible molecular mechanisms that may control immunity and autoimmunity. These findings provide us with new opportunities for targeted, therapeutic modulation of the immune response. Comparing the most significant up- or down-regulated genes, we observed that most of the genes are related to MAPK, PI3K-Akt, FoxO, and NF-kappa B signaling pathway (Gadd45a, Il12b, IL10, Igf1, Il1r1, PIK3R1). In these genes, PIK3R1 is located at the central node of miRNA–mRNA-pathway-net which indicated that it had a strong regulatory effect in the process of lymphocytes stimulated by Kyn. The immune system is highly sensitive to manipulation of PI3K signaling pathway. Some researchers (56) have observed that just a two-fold change in PI3K signal activity through Akt is sufficient to regulate lymphocyte homeostasis and induce autoimmunity in mice.

MicroRNAs (miRNAs) are increasingly being identified as key factors in the immune system regulating immune responses. Although miRNA regulation of each target results in only minor changes in gene expression, these small changes can be amplified by miRNA–mRNA-net to affect the cell behavior. These changes can be easily observed in the immune system, where miRNAs modulate many cells’ ultimate fate by developing mature lymphocytes (57–59). Among the differentially expressed miRNAs, mmu-miR-329-3p (up-regulated) and mmu-miR-3066-3p (down-regulated) were most variable after stimulation with Kyn. The integrated analysis of miRNA and mRNA expression revealed that one miRNA targeting several mRNAs, mmu-miR6916-5p, mmu-miR674-5p, mmu-miR34a-5p and mmu-miR155-3p were shown to have more targets. There are no reports on the function of mmu-miR6916-5p, mmu-miR674-5p, and mmu-miR-3066-3p. MicroRNA-329-3p (miR-329-3p) has been studied in many types of human cancer (60). MiR-155 is one of the most studied miRNAs for its multiple roles in the control of the innate and adaptive immune processes. Several studies (61) demonstrated that miR-155 controls differentiation of CD4^+^ T cells into the T helper cell subsets (Th1, Th2 and Th17) (62–64) and that it affects the development of Tregs (65, 66). MiR-155 also regulates CD8^+^ T cells (67, 68) and is vital for normal B cell differentiation and antibody production (63, 64, 69). MiR-155 over-expression can enhance the anti-viral, as well as anti-tumor CD8^+^ T cell responses *in vivo (* 61 *).* On the other hand, reducing the expression of miR-155 may cause downstream cascades and increase the tendency to generate Th2 cells which secrete type 2 (IL-4, IL-5 and IL-10) cytokines (70). Therefore, fully understanding vaccine factors that influence immune response has important implications. It helps direct and rationally design new and more efficacious vaccines or adjuvants with better immunogenicity and safety profiles (32).

To sum up, our data demonstrated that kynurenine, as an immunosuppressive adjuvant, can successfully help the phage vaccine to induce immune tolerance in NOD mice, thus reducing the symptoms of diabetes. As a physiological substance *in vivo*, kynurenine has superior safety as an adjuvant than other exogenous substances in theory. We believe that kynurenine may be used as a novel immunosuppressive adjuvant in autoimmune disease.

## Data Availability Statement

The datasets presented in this study can be found in online repositories. The names of the repository/repositories and accession number(s) can be found below: https://www.ncbi.nlm.nih.gov/, GSE164304 https://www.ncbi.nlm.nih.gov/, GSE165737.

## Ethics Statement

The animal study was reviewed and approved by Institute of Medical Biology, Chinese Academy institutional animal care and conducted.

## Author Contributions

JS and YH designed the experiments. JS, JDS, JL, MW, SJ, XW, and ZL performed the experiments. JS and JDS analyzed data. JS wrote the manuscript. YL, CM, and NH provided important analysis tools. All authors contributed to the article and approved the submitted version.

## Funding

This study was supported by the CAMS Innovation Fund for Medical Sciences (2017-I2M-3-022), the National Natural Science Foundation of China (31600741), the Yunnan Applied Basic Research Projects (2017FB040), the Yunnan Technology Innovation Talent Projects (2017HB096), and Health Reserve talents in Yunnan Province (H-2017009).

## Conflict of Interest

The authors declare that the research was conducted in the absence of any commercial or financial relationships that could be construed as a potential conflict of interest.

## Supplementary Material

Supplementary Figure 1 qRT-PCR results. **(A)** MircoRNAs **(B)** mRNAs.

Supplementary Figure 2 Analysis of microRNA/mRNA expression profiles in spleen lymphocytes of Balb/C mice stimulated with Kyn *in vitro* compared with the negative control group (NC). **(A, C)** Volcano plots show the differentially expressed miRNAs/mRNAs. **(B, D)** Hierarchical clustering presents the correlation of different samples.

Supplementary Figure 3 Immune related miRNA–mRNA-pathway. Global analysis of immune related miRNA and mRNA which were enriched in both up and down pathways.

Supplementary Table 1 The primers sequence used for q-PCR.

Supplementary Table 2 Differentially expressed miRNA result.

Supplementary Table 3 Differentially expressed mRNA result.

Supplementary Table 4 Gene Ontology analysis for the target gene of differentially expressed miRNA.

Supplementary Table 5 KEGG pathway analysis for the target gene of differentially expressed miRNA.

## Abbreviations

T1D, Type 1 diabetes; Kyn, kynurenine; GAD65, 65 kDa glutamic acid decarboxylase; Tregs, regulatory T cells; NOD, non-obese diabetic; AHR, aryl hydrocarbon receptor.
