# Supplementary figures and images for: The Effect of Immunosuppressive Adjuvant Kynurenine on Type 1 Diabetes Vaccine
Source: Front Immunol. 2021 Jul 7;12:681328. doi: 10.3389/fimmu.2021.681328 (PMC8293994; doi:10.3389/fimmu.2021.681328)

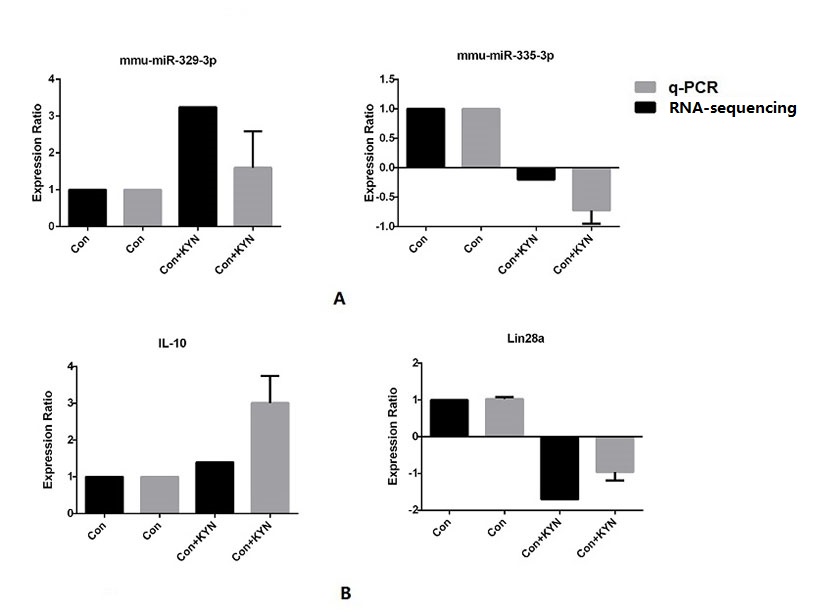

Supplement: Supplementary Figure 1 — qRT-PCR results. (A) MircoRNAs (B) mRNAs. [file Image_1.jpg]

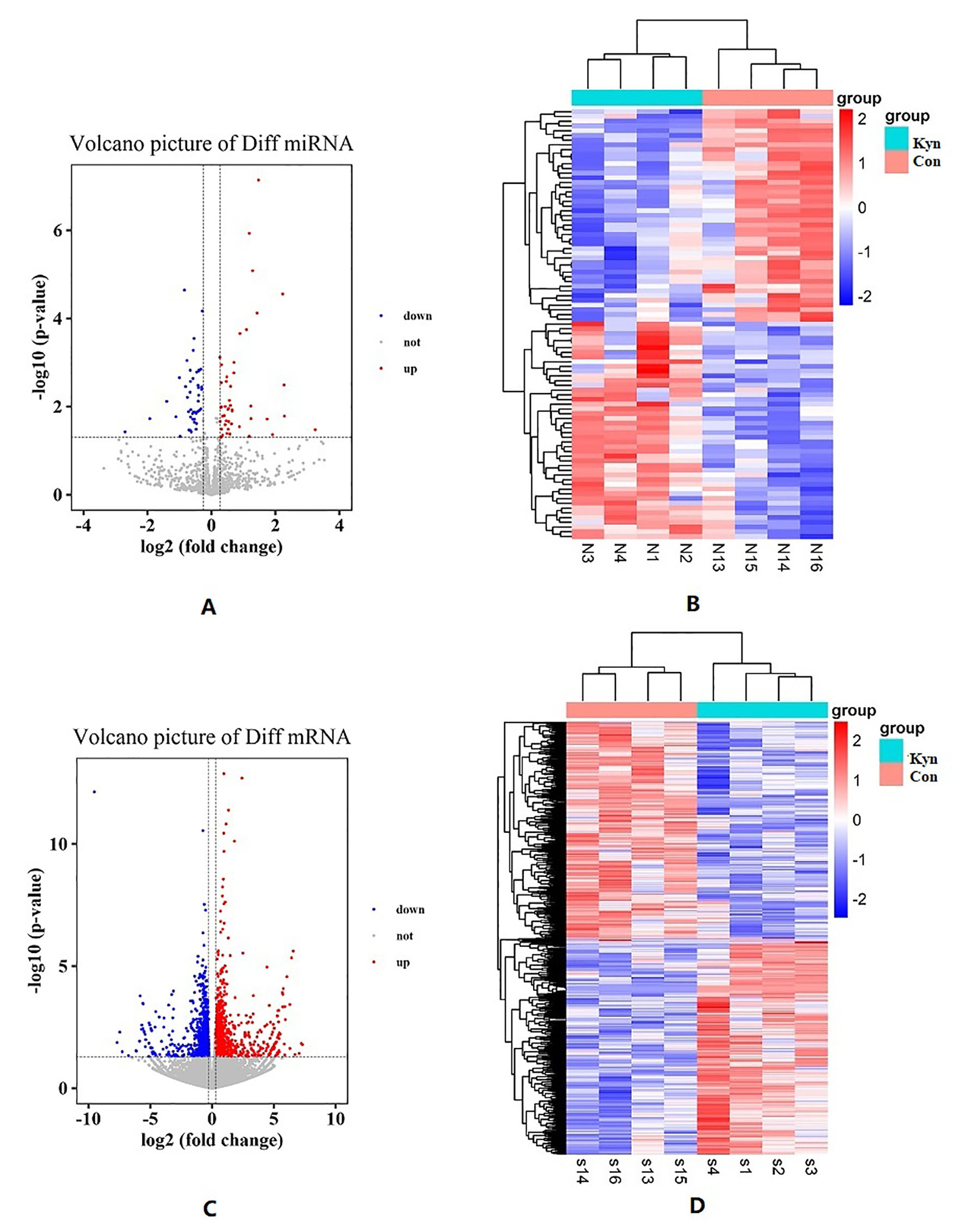

Supplement: Supplementary Figure 2 — Analysis of microRNA/mRNA expression profiles in spleen lymphocytes of Balb/C mice stimulated with Kyn in vitro compared with the negative control group (NC). (A, C) Volcano plots show the differentially expressed miRNAs/mRNAs. (B, D) Hierarchical clustering presents the correlation of different samples. [file Image_2.jpeg]

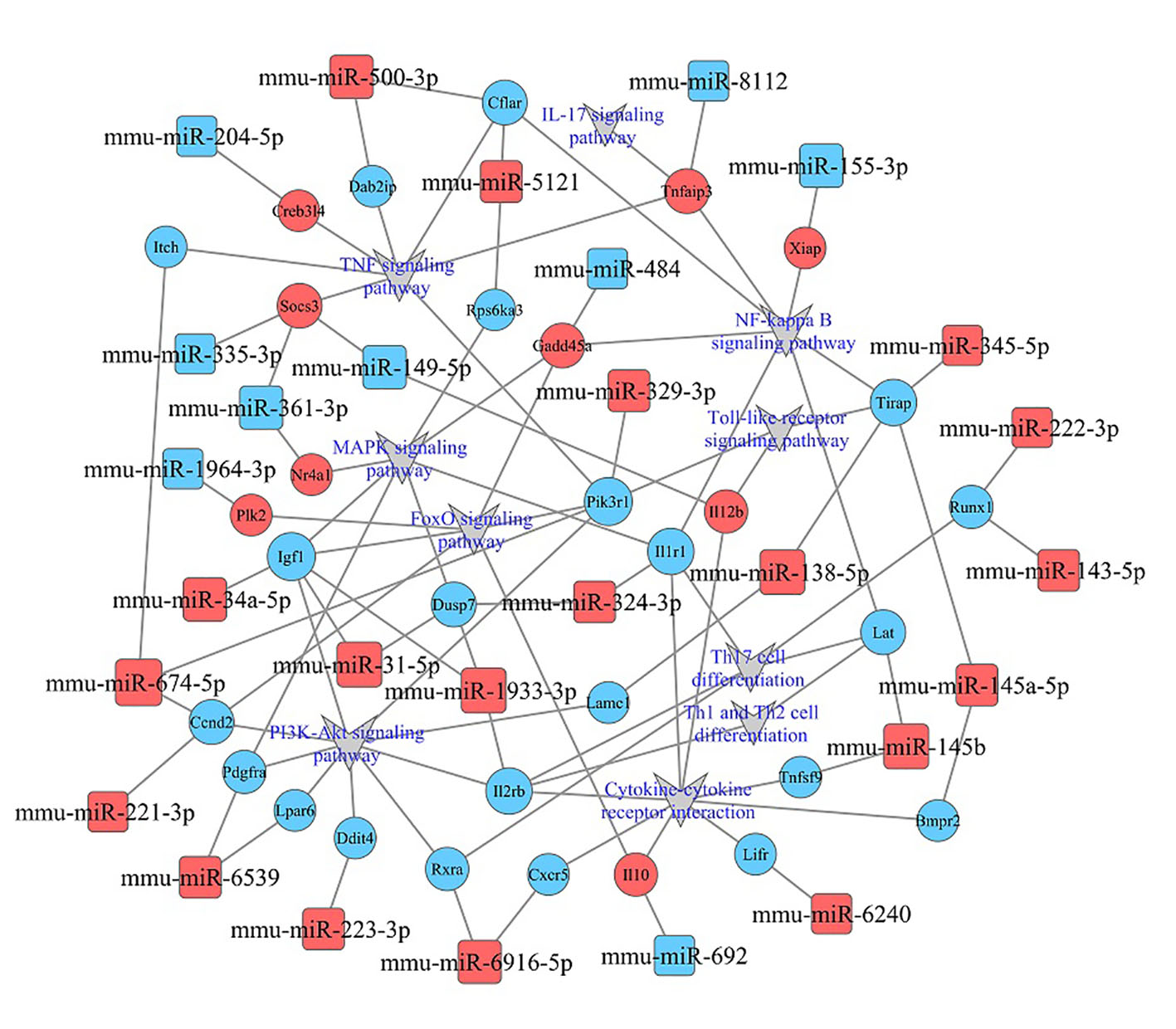

Supplement: Supplementary Figure 3 — Immune related miRNA–mRNA-pathway. Global analysis of immune related miRNA and mRNA which were enriched in both up and down pathways. [file Image_3.jpeg]
